# Data-driven categorization of postoperative delirium symptoms using unsupervised machine learning

**DOI:** 10.3389/fpsyt.2023.1205605

**Published:** 2023-06-27

**Authors:** Panyawut Sri-iesaranusorn, Ryoichi Sadahiro, Syo Murakami, Saho Wada, Ken Shimizu, Teruhiko Yoshida, Kazunori Aoki, Yasuhito Uezono, Hiromichi Matsuoka, Kazushi Ikeda, Junichiro Yoshimoto

**Affiliations:** ^1^Division of Information Science, Nara Institute of Science and Technology, Nara, Japan; ^2^Department of Immune Medicine, National Cancer Center Research Institute, Tokyo, Japan; ^3^Department of Psycho-Oncology, National Cancer Center Hospital, Tokyo, Japan; ^4^Department of Neuropsychiatry, Nippon Medical School, Tama Nagayama Hospital, Tokyo, Japan; ^5^Department of Psycho-Oncology, Cancer Institute Hospital of Japanese Foundation for Cancer Research, Tokyo, Japan; ^6^Department of Clinical Genomics, National Cancer Center Research Institute, Tokyo, Japan; ^7^Department of Pain Control Research, The Jikei University School of Medicine, Tokyo, Japan; ^8^Department of Biomedical Data Science, Fujita Health University School of Medicine, Aichi, Japan

**Keywords:** postoperative delirium, hypothesis-free categorization, K-means clustering, delirium rating scale-revised-98, phenotype, cancer surgery

## Abstract

**Background:**

Phenotyping analysis that includes time course is useful for understanding the mechanisms and clinical management of postoperative delirium. However, postoperative delirium has not been fully phenotyped. Hypothesis-free categorization of heterogeneous symptoms may be useful for understanding the mechanisms underlying delirium, although evidence is currently lacking. Therefore, we aimed to explore the phenotypes of postoperative delirium following invasive cancer surgery using a data-driven approach with minimal prior knowledge.

**Methods:**

We recruited patients who underwent elective invasive cancer resection. After surgery, participants completed 5 consecutive days of delirium assessments using the Delirium Rating Scale-Revised-98 (DRS-R-98) severity scale. We categorized 65 (13 questionnaire items/day × 5 days) dimensional DRS-R-98 scores using unsupervised machine learning (K-means clustering) to derive a small set of grouped features representing distinct symptoms across all participants. We then reapplied K-means clustering to this set of grouped features to delineate multiple clusters of delirium symptoms.

**Results:**

Participants were 286 patients, of whom 91 developed delirium defined according to Diagnostic and Statistical Manual of Mental Disorders, Fifth Edition, criteria. Following the first K-means clustering, we derived four grouped symptom features: (1) mixed motor, (2) cognitive and higher-order thinking domain with perceptual disturbance and thought content abnormalities, (3) acute and temporal response, and (4) sleep–wake cycle disturbance. Subsequent K-means clustering permitted classification of participants into seven subgroups: (i) cognitive and higher-order thinking domain dominant delirium, (ii) prolonged delirium, (iii) acute and brief delirium, (iv) subsyndromal delirium-enriched, (v) subsyndromal delirium-enriched with insomnia, (vi) insomnia, and (vii) fit.

**Conclusion:**

We found that patients who have undergone invasive cancer resection can be delineated using unsupervised machine learning into three delirium clusters, two subsyndromal delirium clusters, and an insomnia cluster. Validation of clusters and research into the pathophysiology underlying each cluster will help to elucidate the mechanisms of postoperative delirium after invasive cancer surgery.

## 1. Introduction

Delirium encompasses a variety of symptoms and clinical effects, such as attention disorders, visual hallucinations, thought disorders, disorientation that impairs communication, visuospatial cognitive impairments that contribute to falls and tumbles, motor agitation that threatens medical safety, and motor inhibition that impairs self-care, delays rehabilitation after surgery, and burdens healthcare workers (1–6). Previous work has shown that delirium is a constellation of various symptoms and trajectories that require further investigation to determine a clear etiology and maximize potential reversibility (7, 8).

Although numerous hypotheses have been proposed for the pathogenesis of delirium, the pathophysiology underlying delirium onset has not yet been clarified. Delirium is a psychiatric condition characterized by a wide variety of psychiatric symptoms. Similar to other psychiatric disorders, phenotypic heterogeneity likely plays an important role in delirium pathogenesis. Previous studies (9–15) have assessed delirium symptoms in detail using the Delirium Rating Scale-Revised-98 (DRS-R-98) and introduced three core domains (cognitive, higher-order thinking, and circadian domains) with accessory symptoms, and four motor subtypes (hyperactive, hypoactive, mixed, and normoactive delirium). The cognitive domain comprises orientation, attention, short- and long-term memory, and visuospatial ability; the higher-order thinking domain consists of language and thought processes; the circadian domain comprises the sleep–wake cycle and motor activity alterations.

Meagher et al. have explored delirium clinical subtyping based on motor symptoms (8, 15–18). Hyperactive delirium is characterized by an increase in motor activity and often results in disruptive or potentially harmful behaviors that jeopardize medical compliance and safety. In contrast, hypoactive delirium is characterized by decreased motor activity. Patients who have both features of increased and decreased activity during an episode are categorized as having the mixed delirium subtype. These three main subtypes are currently used for the diagnosis, prevention, and treatment of delirium in older adults (8, 16, 19); however, these motor subtypes do not specify the trajectory of delirium, nor do they address symptoms other than motility.

Trepacz et al. investigated the influence of age and sex, as well as differences in physical illness, on delirium phenotypes (10). They performed factor analysis and found that delirium had a consistent two-factor structure for delirium phenomenology regardless of age and sex. They also identified the following tendencies: The relationship between the core domains and accessory symptoms was similar in younger patients and women, whereas in older adults and men, accessory symptoms were significantly correlated with the circadian domain but not with other core domains. However, in this previous study, the 13-item severity scale of the DRS-R-98 was reduced to three-dimensional composite scores representing the three core domains of delirium. Although this procedure follows the three-core-domain hypothesis and helps to reduce variability in the factor analysis, it is possible that it ignores the relationship among the original 13-item scales, which could contribute to differences in etiology. Thus, a hypothesis-free, data-driven approach could be useful for exploring new possibilities beyond existing hypotheses.

Postoperative delirium remains to be fully phenotyped. The incidence of postoperative delirium has been reported to range from 11.5 to 50%, with a particularly high incidence in highly invasive cancer surgery (20–23). Measures to prevent postoperative delirium have been investigated (24) because systemic inflammation and hypoxia are known to be mechanistically involved in postoperative delirium (25). These responses are triggered by the wound and anesthesia in relatively fit patients undergoing surgery. Phenotyping postoperative delirium could help in the development of methods of prevention and care. However, to our knowledge, only one study has explored the trajectory of delirium severity after cancer resection, and this study did not consider a variety of phenotypes or assess delirium on consecutive days (26). The phenotypic characteristics of postoperative delirium after invasive cancer resection remain to be elucidated. Furthermore, although categorization techniques using time series datasets have been proposed as methods to better understand the heterogeneity of mental illness (27), they have not been adequately examined for delirium.

In the present study, we aimed to explore the hypothesis-free categorization of patients according to delirium symptoms using an unsupervised machine-learning approach (i.e., an AI technique to find regularity embedded in a data set without desired outputs) to offer new insights into delirium heterogeneity following invasive cancer resection. To identify potential delirium symptom clusters, we applied an unsupervised machine-learning method called K-means clustering to scores obtained from DRS-R-98 assessment over 5 consecutive days after invasive cancer resection.

## 2. Materials and methods

### 2.1. Participants

This was a prospective cohort study to measure postoperative delirium symptoms following invasive cancer resection. The present study was conducted in accordance with the Declaration of Helsinki. Participants were recruited from the National Cancer Center Hospital in Japan, and the study was approved by the Institutional Review Board of the National Cancer Center Japan (2017-282, approved on the 27th of March, 2018). Inclusion criteria were patients undergoing invasive cancer resection, which was defined as an operation planned for 6 h or more with postoperative recovery in the intensive care unit; aged 20 years or older; and who provided written informed consent for participation. Participants who were diagnosed with delirium according to the Diagnostic and Statistical Manual of Mental Disorders, Fifth Edition (DSM-5) (28), at enrollment or 1 day before surgery were excluded from the study. All patients were supported for early mobilization by ICU nurses from postoperative day 1.

### 2.2. Study design

All participants were assessed in the intensive care unit within 2 h of surgery to assess the emergence of delirium according to the DSM-5 (denoted as Day 0) and between 12:00–17:00 on each of the 5 consecutive postoperative days (denoted as Days 1 to 5). In addition, postoperative assessments were performed using the Japanese version of the DRS-R-98 severity scale (20, 29), which assesses delirium severity on 13 items. We defined delirium as positive according to the DSM-5 but used the DRS-R-98 severity scale to investigate various symptoms of delirium following invasive cancer resection and to categorize participants. Diagnostic characteristics were not considered; rather, we focused on symptoms. As an exclusion criterion, positive delirium according to the DSM-5 1 day before surgery enabled us to determine the onset of delirium. All available clinical information was used for the assessment of delirium according to the DSM-5 and DRS-R-98 severity scale. This included witness accounts from nurses and families and recorded assessments using the Confusion Assessment Method for the Intensive Care Unit (30) and the Nursing Delirium Screening Scale (31).

To assess the risk factors for the postoperative delirium cluster, as reported in previous studies (32, 33), we assessed participants’ cognitive function at recruitment using the Mini-Mental State Examination (MMSE) (34) and preoperative anxiety at baseline using the Hospital Anxiety and Depression Scale-Anxiety (HADS-A) (35). We used interviews and medical records to obtain additional information, including daily preoperative use of benzodiazepines (36), anesthesia type (37), and duration of surgery (38) (Supplementary Tables 1, 2).

### 2.3. Data analysis

#### 2.3.1. Unsupervised K-mean clustering

The K-means clustering algorithm is an unsupervised learning algorithm used to classify a given set of data into K distinct clusters/groups so that internal cohesion within the clusters is optimized. It is widely used in various biomedical fields, such as gene expression analysis, disease prediction, and psychological investigations (further details are provided in Supplementary Methods 1).

#### 2.3.2. Feature grouping and dimension reduction

The matrix included 65 (13 items/day × 5 days) DRS-R-98 score entries (Supplementary Figure 1). To perform hypothesis-free extraction of factors representing core symptoms from all features, we used latent variable modeling and derived factor scores for each participant. Specifically, derivation was implemented using the K-means clustering method (39, 40) to partition all features into different clusters, and a collection of averaged values within the clusters was used as a low-dimensional feature vector in subsequent analyses.

#### 2.3.3. Identification of the participant cluster

To identify the cluster structure of the participants in a data-driven manner, we reapplied the K-means clustering algorithm to the low-dimensional feature vector dataset described in the previous section (further details are provided in Supplementary Methods 2). Of note, this patient clustering was based on delirium-related symptoms during the 5-day postoperative period, grouped in a hypothesis-free manner, and did not lead to a predictive model of patient trajectory.

To characterize the resulting clusters, we also performed *post hoc* group comparison among the participant clusters using various scores associated with background characteristics and the previously mentioned low-dimensional feature vector. The complete data analysis workflow is summarized in Supplementary Figure 2.

#### 2.3.4. Determination of K for the K-means clustering

Determining the optimal number of clusters (ordinarily denoted as *K*) is an important aspect of K-means clustering algorithms. In this study, the Akaike information criterion (41, 42) was used as the primary criterion for both dimension reduction and participant clustering (further details are provided in Supplementary Methods 2).

## 3. Results

Of the 384 consecutively recruited eligible patients, 3 were excluded because of delirium at recruitment and 54 declined to participate, resulting in a total of 327 study participants. The analyses were performed on 286 patients (74.5% of the recruited population) who underwent invasive cancer resection and completed 5 consecutive days of delirium assessments (Supplementary Figure 3). Ninety-one patients were diagnosed with postoperative delirium according to the DSM-5 (Table 1). The delirium group was older, less likely to be working, had poorer cognitive function, had longer surgery and anesthesia duration, and exhibited more severe delirium symptoms as assessed using the DRS-R-98 (Table 1). The characteristics of these variables were analyzed for the identified participant clusters.

**Table 1:** Background characteristics of the study participants.

	All	Delirium	Non-delirium	*p*-value
Study participants (*n*)	286	91	195	
*Resection cancer site*				
Esophagus	100	18	82	
Hepatobiliary and pancreas	103	42	61	
Head and Neck	72	29	43	
Duodenum	11	2	9	
*Preoperative factors*				
Age (years)	65.9 ± 0.6	71.2 ± 0.7	63.4 ± 0.8	<0.001*
Male (ratio)	73.8%	81.3%	70.3%	0.06
BMI (kg/m^2^)	22.5 ± 0.2	22.3 ± 0.3	22.6 ± 0.2	0.445
Education >12 years (ratio)	53.5%	45.1%	57.4%	0.0568
Single (ratio)	21.7%	19.8%	22.6%	0.705
Employment status: currently working (ratio)	61.9%	46.2%	69.2%	<0.001*
Performance status >0 (ratio)	10.5%	13.2%	9.2%	0.418
CCI >0 (ratio)	50.0%	60.4%	45.1%	0.0223*
ASA-PS >2 (ratio)	15.4%	19.8%	13.3%	0.218
CAGE >2 (ratio)	10.5%	28.6%	23.6%	0.418
History of delirium (ratio)	9.4%	11.0%	8.7%	0.693
History of psychiatric disorder (ratio)	5.6%	6.6%	5.1%	0.838
Benzodiazepine use (ratio)	7.7%	13.2%	5.1%	0.032*
Antipsychotic use (ratio)	2.1%	3.0%	1.5%	0.601
Steroid use (ratio)	2.8%	3.3%	2.6%	1
Opioid use (ratio)	3.5%	4.4%	3.1%	0.826
Preoperative MMSE (score)	28.5 ± 0.1	27.9 ± 0.2	28.7 ± 0.1	<0.001*
Preoperative HADS-A > 7 (ratio)	21.0%	24.2%	20.1%	0.531
*Intraoperative factors*				
Duration of surgery (h)	6.8 ± 0.1	7.1 ± 0.2	6.7 ± 0.1	0.0192*
Duration of anesthesia (h)	8.0 ± 0.1	8.3 ± 0.2	7.8 ± 0.1	0.0242*
Blood loss (ml)	575.0 ± 49.0	682.9 ± 87.0	524.7 ± 59.1	0.134
Total intravenous anesthesia (ratio)	12.2%	4.4%	15.9%	0.0102*
Severity of delirium symptoms				
DRS-R-98 max (score)	5.69 ± 0.3	12.53 ± 0.6	2.5 ± 0.1	<0.001*

The *p*-values were evaluated using chi-square tests and two-sample *t*-tests (*p < 0.05) for continuous variables. The representative values and errors of the continuous variables are the averages and the standard errors of the mean, respectively. ASA-PS, American Society of Anesthesiologists physical status; BMI, body mass index; CCI, Charlson’s Comorbidity Index; CAGE, CAGE questionnaire, Cut, Annoyed, Guilty, Eye-opener, a screening test for potential alcohol problems; MMSE, Mini-mental state examination; HADS-A, Hospital anxiety and depression scale-anxiety; DRS-R-98 max, maximum score of the Delirium Rating Scale-Revised-98 severity scale during the 5 postoperative days.

### 3.1. Feature grouping

**Figure 1:**
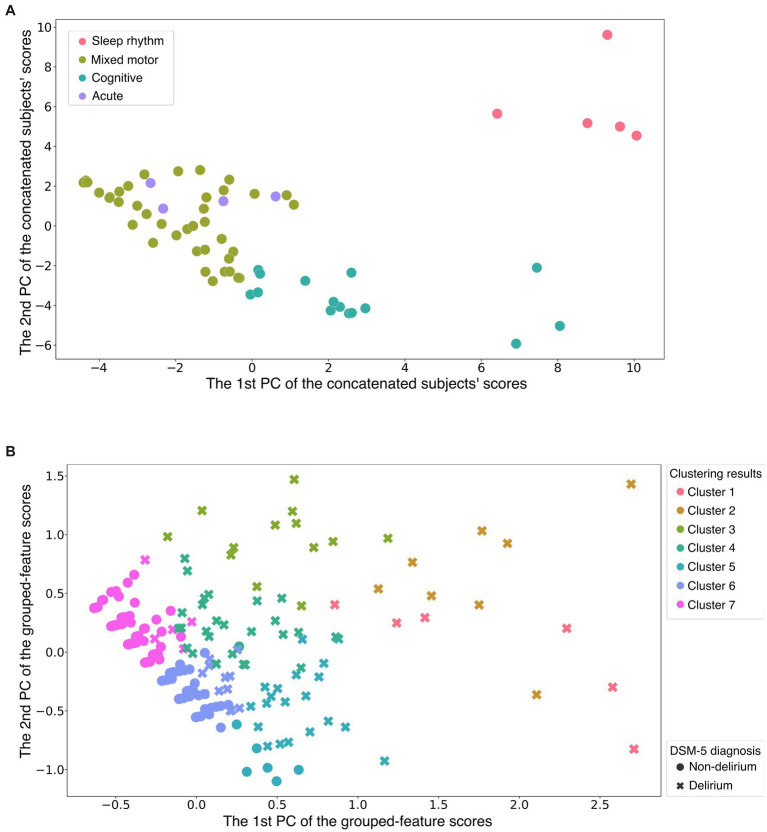
Two-dimensional visualization of feature grouping and participant clustering. **(A)** The horizontal and vertical axes are the first and second principal component scores of the column vectors of the original data matrix, respectively (Supplementary Figure 2 shows how to create the figure). Each dot indicates a single feature (a DRS-R-98 severity item on a specific day). Color difference shows group difference determined by the first K-means clustering. This suggests that all features assigned to the same group were quite similar to each other. The alternative visualization using the heatmap is shown in Supplementary Figure 8. **(B)** The horizontal and vertical axes are the first and second principal component scores of the row vectors of the data matrix after dimension reduction processing, respectively. Each point indicates a single participant; the color difference shows the cluster difference determined by the second K-means clustering. Two different markers of the points (crosses and filled circles) indicate whether the corresponding participants were diagnosed as delirium or non-delirium. Participants who belonged to the non-delirium cluster were distributed closely together, whereas the delirium participants were distributed widely. The alternative visualization of participant clusters using the heatmap is shown in Supplementary Figure 9. Sleep rhythm = sleep–wake cycle disturbance; Cognitive = cognitive and higher-order thinking domain with perceptual disturbance and thought content abnormalities; Acute = acute and temporal response; DSM-5 = Diagnostic and Statistical Manual of Mental Disorders, Fifth Edition; PC = principal component (see also Figure 2).

**Figure 2:**
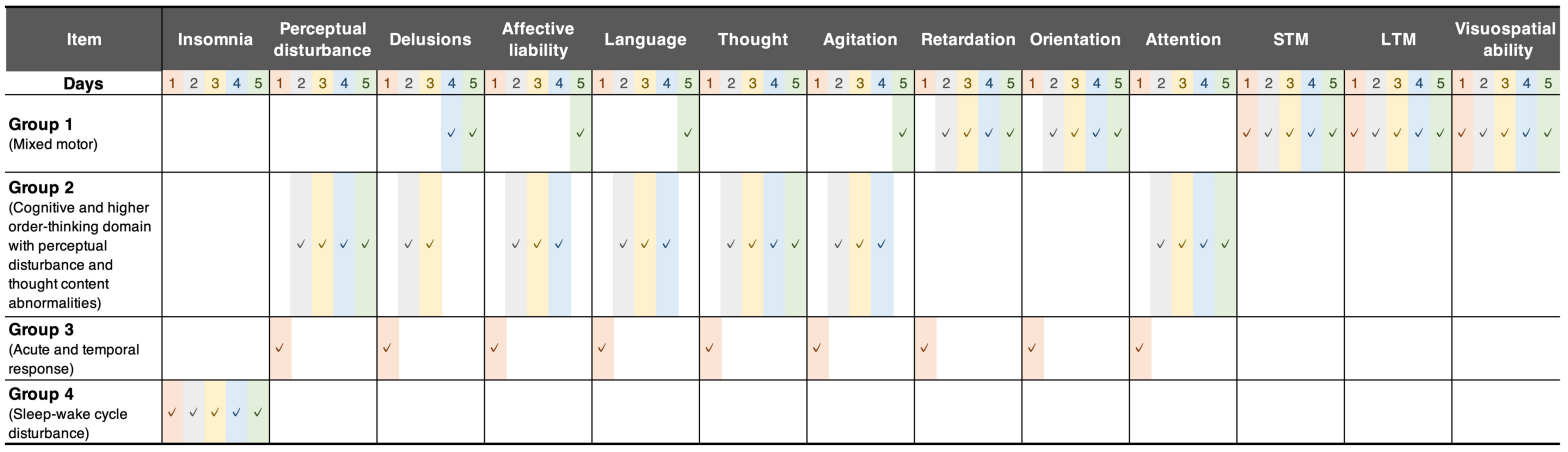
Grouped features. These groups were derived from the first K-means clustering. The checkmarks indicate the group assignment of each feature. Group 1 comprised initial motor retardation with cognitive items and then the trajectory changed to affective lability, motor hyperactivity, and delusion. Group 2 consisted mainly of cognition, language, and thought processes with perceptual disturbances and abnormal thought content after postoperative Day 2. Group 3 indicated temporal response until postoperative Day 1, including the emergence of delirium. Group 4 comprised only sleep–wake cycle disturbance during postoperative 5 days. STM, short-term memory; LTM, long-term memory.

We first performed feature grouping of the original (286 × 65-dimensional) data matrix using the K-means clustering algorithm. To avoid suboptimal local minima intrinsic to the K-means clustering algorithm, we executed the algorithm 1,000 times with a different initialization for each *K* and subsequently selected the clustering solution with the lowest Akaike information criterion score as the representative result (Supplementary Figures 4–7). The results showed four feature groups, as shown in Figure 1A (the clustering result is provided in Supplementary Figure 8). Following visual inspection, each feature group was interpreted as follows (Figure 2):

- Mixed motor: This group comprised initial motor retardation with cognitive items and then the trajectory changed to affective lability, motor hyperactivity, and delusion.
- Cognitive and higher-order thinking domain with perceptual disturbance and thought content abnormalities: This group was mainly characterized by cognition, language, and thought processes with perceptual disturbances and abnormal thought content after postoperative Day 2. Of note, it is also characterized by agitated behavior feature on postoperative Days 2–4.
- Acute and temporal response: This group indicated a temporal response after postoperative Day 1 following surgery including emergence delirium.
- Sleep–wake cycle disturbance: Insomnia-related features were grouped independently from other features.

### 3.2. Participant clustering

As shown in Section 3.1, we extracted four feature groups using K-means clustering. By calculating the average score for each feature group, we transformed the original data matrix *X* into the dimension-reduced (286 × 4-dimensional) matrix *X*. By applying K-means clustering to *X*, we found that the 286 participants could be divided into seven clusters (See the validity of the number of clusters in Supplementary Figures 4–7). Figure 1B illustrates the clusters in reduced two-dimensional space after applying principal component analysis, and shows that participants who belonged to the non-delirium cluster were distributed closely together. In contrast, the delirium participants were distributed widely in the two-dimensional space.

Table 2 shows the demographic and clinical attributes of the participants assigned to each cluster, the order of which was sorted in descending order of the within-cluster average of the maximum DRS-R-98 score over the 5 postoperative days as a measure of delirium symptom severity. Based on the threshold between the number of delirium and non-delirium participants in each cluster, clusters 1 to 5 were treated as delirium clusters, and clusters 6 and 7 were considered non-delirium clusters. Additionally, we used analysis of variance to test the hypothesis that there were no differences between any of the clusters. Our results showed significant differences between two or more clusters for the features of age (*p* < 0.001), baseline MMSE score (*p* < 0.005), and the emergence of delirium (*p* < 0.001). To examine the distribution of each cluster for each grouped feature, we used heatmap visualization, as shown in Supplementary Figure 9. We also generated a radar chart to visualize the mean of each cluster for each of the 13 feature types (Supplementary Figure 10). That clarified that non-delirium participants showed very low scores for all items, whereas delirium participants showed substantial or large scores for one or more items, and the radar shapes varied among delirium clusters.

**Table 2:** *Post hoc* analysis for the characteristics of participant clusters.

Cluster	Number of participants (n)		Age (years)	Education >12 years (ratio)	Currently working (ratio)	CCI >0 (ratio)	Operative duration (min)	BZD use (ratio)	TIVA (ratio)	Preoperative MMSE (score)	Pre-operative HADS-A (score)	Emergence delirium (ratio)	DRS-R-98 max (score)
	Delirium (male /female)	Non-delirium (male /female)											
1	6 (5/1)	0	70.8 ± 1.2	16.7%	50.0%	50.0%	440.8 ± 14.2	16.7%	0.0%	26.8 ± 1.1	4.5 ± 1.5	16.7%	**25.2 ± 1.5**
2	8 (5/3)	0	**75.6 ± 2.1**	25.0%	12.5%	50.0%	**464.6 ± 37.7**	12.5%	12.5%	27.1 ± 0.5	**7.5 ± 0.9**	62.5%	20.8 ± 1.3
3	13 (12/1)	0	68.5 ± 2.1	**61.6%**	46.2%	**76.9%**	435.3 ± 28.6	**23.1%**	7.7%	27.6 ± 0.7	5.8 ± 0.9	**69.2%**	14.6 ± 0.9
4	27 (20/7)	1 (1/0)	73.1 ± 1.0	39.3%	32.1%	53.6%	419.5 ± 17.1	14.3%	3.6%	27.8 ± 0.5	5.2 ± 0.7	7.1%	10.5 ± 0.7
5	20 (17/3)	6 (3/3)	70.3 ± 1.3	50.0%	57.7%	62.5%	419.2 ± 15.2	11.5%	11.5%	28.4 ± 0.4	6.0 ± 0.8	15.4%	9.2 ± 0.6
6	12 (11/1)	68 (52/16)	63.4 ± 1.3	61.3%	**62.5%**	51.3%	391.5 ± 11.3	6.3%	11.3%	**28.8 ± 0.2**	5.4 ± 0.4	3.8%	3.7 ± 0.3
7	5 (4/1)	120 (81/39)	63.9 ± 0.9	55.2%	60.8%	43.2%	406.7 ± 9.9	4.0%	**16.0%**	28.7 ± 0.1	4.9 ± 0.3	0.8%	2.3 ± 0.2
Total	91 (74/17)	195 (137/58)	65.9 ± 0.6	53.5%	55.9%	50.0%	408.5 ± 6.1	7.7%	12.2%	28.5 ± 0.1	5.3 ± 0.2	8.7%	5.7 ± 0.3

The bold and underlined numbers represent the maximum and minimum values of each feature, respectively. The representative values and errors of continuous variables are the averages and the standard errors of the mean, respectively. CCI, Charlson’s Comorbidity Index; BZD use, regular preoperative use of benzodiazepines; TIVA, total intravenous anesthesia with propofol; MMSE, mini-mental state examination; HADS-A, Hospital anxiety and depression scale-anxiety; DRS-R-98 max, maximum score of the Delirium Rating Scale-Revised-98 during the 5 postoperative days.

### 3.3. Cluster characteristics

To determine how the association between the feature groups and participant clusters should be interpreted, we performed *post hoc* analyses using one-sample *t*-tests. The null hypothesis was that the population average in a cluster would be equal to the overall average of all participants for each grouped feature (baseline). We used Bonferroni correction to correct for multiple tests, and set the significance level to a family-wise error rate of 0.05. Table 3 shows the attributes of all seven clusters based on the *post hoc* tests. The characteristics of each cluster are as follows:

- Cognitive and higher-order thinking domain dominant delirium: This cluster had a feature of “cognitive and higher-order thinking domain with perceptual disturbance and thought content abnormalities” that was significantly higher than baseline.
- Prolonged delirium: This cluster had “mixed motor,” “cognitive and higher-order thinking domain with perceptual disturbance and thought content abnormalities,” and “acute and temporal response” features that were significantly higher than baseline.
- Acute and brief delirium: This cluster is characterized solely by an “acute and temporal response” that is significantly higher than the baseline.
- Subsyndromal delirium-enriched: This cluster had both features of “mixed motor” and “cognitive and higher-order thinking domain with perceptual disturbance and thought content abnormalities” that were significantly higher than baseline.
- Subsyndromal delirium-enriched with insomnia: This cluster had “cognitive and higher-order thinking domain with perceptual disturbance and thought content abnormalities” and “sleep rhythm” features that were significantly higher than baseline.
- Insomnia: This cluster had a “sleep rhythm” feature that was significantly higher than baseline, as well as “mixed motor,” “cognitive and higher-order thinking domain with perceptual disturbance and thought content abnormalities,” and “acute and temporal response” features that were significantly lower than baseline.
- Fit: All features in this cluster were significantly lower than the baseline.

**Table 3:** Clinical interpretations of the participant clusters.

Cluster	Mixed motor	Cognitive	Acute and temporal response	Sleep–wake cycle disturbance	Clinical interpretation
1	N.S.	+ + +	N.S.	N.S.	Cognitive and higher-order thinking domain dominant delirium
2	+	+ +	+ + +	N.S.	Prolonged delirium
3	N.S.	N.S.	+ + +	N.S.	Acute and brief delirium
4	+ + +	+ + +	N.S.	N.S.	Subsyndromal delirium-enriched
5	N.S.	+ +	N.S.	+ + +	Subsyndromal delirium-enriched with insomnia
6	− − −	− − −	− − −	+ + +	Insomnia
7	− − −	− − −	− − −	− − −	Fit

*The plu*s (+) and minus symbols (−) indicate that the population average in a cluster was higher and lower, respectively, than the ground average of all participants: + or −: p<0.05; + + or − −: p<0.01; + + + or − − −: p<0.001; N.S.: p≥0.05. The *p*-values were calculated using two-sample *t*-tests, uncorrected for multiple comparisons. Cognitive: Cognitive and higher-order thinking domain with perceptual disturbance and thought content abnormalities.

## 4. Discussion

This is the first study to classify patients by delirium symptoms using hypothesis-free classification with K-means clustering. Although machine-learning approaches have been widely used in psychiatry (43), this study demonstrated their potential utility in the development of delirium research. The K-means clustering analysis of the DRS-R-98 assessment identified four features of delirium symptoms. It was particularly notable that the acute and temporal response feature group was the only group that contained first-day features, whereas the sleep–wake cycle disturbance feature group contained only features from the insomnia item. The participant clustering analysis produced seven clusters with three delirium clusters and two subsyndromal delirium clusters, whereas previous studies have posited only three subtypes of hyperactive delirium, hypoactive delirium, and mixed delirium (11). Although our findings included motor-related features, in line with previous research, we also found additional clusters based on the grouped features. For example, only participants in the subsyndromal delirium-enriched with insomnia cluster and the insomnia cluster had a significantly high value for the sleep rhythm feature. Although these clusters indicated low delirium severity with sleep–wake rhythm disturbance in the circadian domain, they may be a characteristic delirium-related symptom following highly invasive cancer surgery. Drugs to prevent delirium by targeting sleep–wake rhythm disturbances are being developed, of which melatonin, the melatonin analog ramelteon (44, 45), and orexin-receptor antagonists (45, 46) are potential candidates that may also be effective for patients in the subsyndromal delirium-enriched with insomnia cluster following highly invasive cancer surgery. It is also notable that the time course could be divided into an acute and brief delirium cluster and a prolonged delirium cluster. Procedures and complications during the recovery process may affect the time course of postoperative delirium. It is well established that some physical reactions peak immediately after cancer surgery, whereas others peak later and are prolonged (47), which may contribute to the difference between clusters.

This study had several limitations. First, we attempted to classify patients following cancer surgery using delirium-related symptoms in a data-driven manner. However, the statistical approach comprised a group-level analysis, not an individual-level analysis as used in previous research by Meagher et al. (15, 48), and thus did not lead to a predictive model of delirium symptoms. It is unclear whether the clusters found in this study are useful for clinical prediction; for example, the present findings do not suggest that having insomnia the day after surgery predicts insomnia on the fifth postoperative day. However, these findings show that (1) insomnia during 5 postoperative days is more likely to be linked to other delirium symptoms, and (2) sleep rhythm disturbances are a notable feature of the hypothesis-free classification of postoperative patients. Additionally, we did not cluster postoperative delirium patients; rather, we clustered postoperative patients according to delirium symptoms. After validation, this group-level analysis will allow comparison between groups and may help to elucidate the delirium mechanism, taking into account its heterogeneity.

Second, with regard to the data collection process, when we assessed delirium, we could not control the duration between the surgery and the Day 1 assessment. Delirium symptoms fluctuate and tend to be worse at night. Therefore, although performing assessments at night can provide more accurate delirium data, participants are less cooperative than in the daytime. Given this issue, we assessed delirium symptoms from 12:00 to 17:00 using the DRS-R-98 using information from a medical record. This included assessment data from the Confusion Assessment Method for the Intensive Care Unit (30) and the Nursing Delirium Screening Scale (31) obtained from 17:00 the day before the assessment or surgery. Moreover, we used witness accounts of nurses and families to obtain information about patient symptoms until the assessments were performed.

Third, we collected data during the 5 days after surgery and did not analyze symptoms on postoperative Day 6 or later, which may have resulted in missing data for some aspects of delirium. However, our previous study showed that most delirium features manifest by postoperative Day 4 following invasive cancer surgery (49). Therefore, in line with another study (50), we consider that assessing delirium during the 5 days after surgery is appropriate because a longer assessment period increases the probability of delirium being triggered by factors other than surgery.

The fourth limitation is related to the nature of the K-means clustering algorithm. Although the algorithm can be used without prior knowledge of the categorization of features and participants, solutions generally depend on the initial setup and the assumed number of clusters. Thus, it is not feasible to determine a theoretically optimal clustering solution. However, to minimize the dependence, we executed the algorithm 1,000 times, with different initializations for each number of clusters, which varied considerably from 1 to 10. Because we selected the optimal number from all 10,000 clustering solutions based on the Akaike information criterion, our result has high reproducibility as long as the same dataset is analyzed. In addition, we used two-step clustering, in which both DRS-R-98 questionnaire items and participants were categorized in turn, then the items assigned to the same category were summarized using the group average. This aspect of our methodology addresses high within-subject and between-item correlations. In particular, categorization of DRS-R-98 items made it easier to interpret the properties of each cluster (as shown in Figure 2; Supplementary Figure 9).

In related work, Meagher et al. used the generalized estimating equations to analyze the relationship between motor subtypes in delirium and longitudinal phenomenological symptoms assessed by DRS-R-98 (15). This approach is very helpful in characterizing specific subtypes by delirium-related symptoms because it can consider the correlation of within-subject data, which is intrinsic to the type of longitudinal data we collected. However, the generalized estimating equation method itself is not capable of discovering unknown subtypes. Therefore, potential subtypes should be defined based on prior knowledge before applying generalized estimating equations. As the objective of this study was to perform hypothesis-free phenotyping rather than characterize existing subtypes, we used unsupervised K-means clustering. However, a disadvantage of this method is that we had to compromise the nature of the longitudinal data by flattening it to a vector, even though the correlation of within-subject data was partly taken into account by the feature grouping process. We plan to conduct future studies to develop methods to overcome this limitation.

Furthermore, owing to the nature of the data-driven approach, the optimal partition of features can depend on the dataset; thus, we should also investigate whether the structure of the feature grouping is stable over other datasets.

The final limitation is the generalizability of our results to other datasets. The clustering we used in this study constitutes explanatory data analysis to identify a plausible hypothesis. The current findings must now be validated and reproduced using other datasets, especially those that include different anesthesia approaches, cancer sites, and data collection processes. Future validation studies are needed to address these issues.

## 5. Conclusion

In this study, we used unsupervised machine learning to identify a new phenotype delirium group based on 13 delirium symptoms after invasive cancer surgery. Our data demonstrated seven subgroups of patients with delirium symptoms following surgery: (i) cognitive and higher-order thinking domain dominant delirium, (ii) prolonged delirium, (iii) acute and brief delirium, (iv) subsyndromal delirium-enriched, (v) subsyndromal delirium-enriched with insomnia, (vi) insomnia, and (vii) fit. Future studies should apply these procedures to other participant datasets for validation and to explore the etiology of delirium.

## Data availability statement

The original contributions presented in the study are included in the article/Supplementary material, further inquiries can be directed to the corresponding authors.

## Ethics statement

The studies involving human participants were reviewed and approved by Institutional Review Board of the National Cancer Center Japan (2017-282). The patients/participants provided their written informed consent to participate in this study.

## Author contributions

RS, JY, TY, KA, and YU contributed to the conception and design of the study. RS, SM, SW, and KS collected clinical information and organized the database. PS and JY performed the data analysis and wrote the first draft of the manuscript. RS wrote sections of the manuscript. All authors contributed to the article and approved the submitted version.

## Funding

This study was supported by a JSPS KAKENHI grant (no. 18K18468) and the Practical Research for Innovative Cancer Control Project (no. 18ck0106458h0001) from the Japan Agency for Medical Research and Development.

## Conflict of interest

The authors declare that the research was conducted in the absence of any commercial or financial relationships that could be construed as a potential conflict of interest.

## Publisher’s note

All claims expressed in this article are solely those of the authors and do not necessarily represent those of their affiliated organizations, or those of the publisher, the editors and the reviewers. Any product that may be evaluated in this article, or claim that may be made by its manufacturer, is not guaranteed or endorsed by the publisher.

## Abbreviations

DRS-R-98: Delirium rating scale-revised-98
DSM-5: Diagnostic and statistical manual of mental disorders, fifth edition
HADS: Hospital anxiety and depression scale-anxiety
MMSE: Mini-mental state examination
