# Supplementary material for: Data-driven categorization of postoperative delirium symptoms using unsupervised machine learning
Source: Front Psychiatry. 2023 Jun 27;14:1205605. doi: 10.3389/fpsyt.2023.1205605 (PMC10333495; doi:10.3389/fpsyt.2023.1205605)
Supplement: Supplementary file 1 [file Data_Sheet_1.docx]

Supplementary Material

Data-Driven Categorization of Postoperative Delirium Symptoms Using Unsupervised Machine Learning

Panyawut Sri-iesaranusorn, Ryoichi Sadahiro*, Syo Murakami, Saho Wada, Ken Shimizu, Teruhiko Yoshida, Kazunori Aoki, Yasuhito Uezono, Hiromichi Matsuoka, Kazushi Ikeda, and Junichiro Yoshimoto*

*** Correspondence:** Junichiro Yoshimoto: junichiro.yoshimoto@fujita-hu.ac.jp; Ryoichi Sadahiro: rsadahir@ncc.go.jp

# Supplementary methods 1: K-means clustering

The K-means clustering algorithm is an unsupervised learning algorithm used to classify a given set of data into *K* distinct clusters/groups so that internal cohesion within the clusters is optimized (1). It is widely used in various biomedical fields, such as gene expression analysis (2–4), disease prediction (5, 6), and psychological investigations (7, 8). The details of the procedure are as follows: i) the data matrix was rearranged to $Y=X^{T}$, where ^T^ denotes the transpose of the matrix; ii) the K-means clustering algorithm was applied to the data matrix $Y$, and we obtained the result $C_{l}\subseteq\left\{ 1,\ldots, p \right\} (l=1,\ldots,K)$, where $C_{l}$ denotes the subset of feature indices assigned to the *l*-th group; and iii) the dimension-reduced matrix  $\bar{X}\equiv\left[ \bar{x}_{il} \right]$ was constructed, where $x_{il}=\left( \frac{1}{\left| C_{l} \right|} \right)\sum_{j\in C_{l}} x_{ij}$ is the value averaged over the features assigned to the *l*-th group for the *i*-th participant.

Given a dataset $D=\left\{ x_{n} \right\}_{n=1}^{N}$ and a specific number of clusters *K* as the inputs, the algorithm starts by initializing the centroids of *K* clusters at random. Subsequently, the expectation and maximization steps are alternately iterated. In the expectation step, each data point is assigned to the cluster with the closest centroid in terms of a predefined distance measure. To this end, we used Euclidean distance based on the standard manner. In the maximization step, the centroids of the clusters were recalculated and updated so that they agreed with the centroids of the data points assigned to the corresponding clusters. The two-step iterations were repeated until there were no changes to the centroids of the clusters. Further details are available elsewhere (1).

For any predefined *K*, the K-means clustering converges to a cluster assignment to locally minimize the following objective function:

$S_{w}\left( K \right)=\sum_{i=1}^{K} \sum_{x\in C_{i}} \left\| x-\bar{x}_{i} \right\|^{2}$(S1)

where *C_i_* is the set of data points assigned to the *i*-th cluster $\left( i=1,\ldots,K \right)$, and  $\bar{x}_{i}$ is the centroid of the *i*-th cluster. $\left\| x-y \right\|$ denotes the Euclidean distance between two vectors of *x* and *y*. Eq. (S1) is called *distortion* or the *within-cluster sum of squares* and measures the degree of internal cohesion within the clusters (smaller $S_{w}\left( K \right)$ indicates better internal cohesion). The external separation between the clusters is also important for clustering and can be measured using the formula:

$S_{b}\left( K \right)=\sum_{i=1}^{K} \left| C_{i} \right|\left\| \bar{x}_{i}-\bar{x} \right\|^{2} ($S2)

where $\left| C_{i} \right|$ denotes the number of data points assigned to the *i*-th cluster, and *x̄* is the centroid of all data points. Eq. (S2) is called the *between-cluster sum of squares* (larger $S_{b}\left( K \right)$ shows better external separation). $S_{w}\left( K \right)$ and $S_{b}\left( K \right)$ always satisfy the following relationship:

$S_{w}\left( K \right)+S_{b}\left( K \right)=S\left( K \right)=\sum_{n=1}^{N} \left\| x_{n}-\bar{x} \right\|^{2}$(S3)

where $S\left( K \right)$ is the *total sum of squares*. Because the total sum of squares $S\left( K \right)$ is dependent on the dataset but not the cluster assignment, the maximization of the between-cluster sum of squares $S_{b}\left( K \right)$ can be implicitly achieved by minimizing the within-cluster sum of squares $S_{w}\left( K \right)$. In summary, K-means clustering is designed to simultaneously achieve better internal cohesion within the clusters and better external separation between the clusters.

It is worth noting that K-means clustering guarantees convergence to a locally minimal solution but not a globally minimal solution. This suggests that the cluster assignment after convergence depends on the random initialization of the centroids of *K* clusters. To obtain the (approximately) globally optimal solution for each predefined *K*, we performed 1000 K-means clustering runs starting from random initializations and selected the best cluster assignment that achieved the smallest within-cluster sum of squares (Eq. S1) out of all the runs.

# Supplementary methods 2: Determining K for the K-means clustering

Although $S_{w}\left( K \right)$ is a useful measure to improve the cluster assignment for a fixed *K*, it can easily be reduced by increasing *K* (because $S_{w}\left( K \right)=0$ can be achieved by setting $K=N$ and assigning the data points to distinct clusters). Thus, $S_{w}\left( K \right)$ is not a suitable measure to determine the optimal *K*. Although there is no theoretical solution applicable to general situations, several heuristics and criteria have been proposed to determine a “reasonable” value of *K*. Let $K$ and $K^{'}$ be the number of feature clusters and the number of participant clusters, respectively. We varied *K* (and *K'*) from 2 to 10 and evaluated the quality of each using the following four criteria:

- **Elbow method**: This is the most widely used heuristics owing to its simplicity (9, 10). We drew a graph of $S_{w}\left( K \right)$ as a function of *K*, and selected the *K* with the most remarkable curvature that resembled an “elbow.” The selection was performed by visual inspection and was not based on an objective criterion. In the feature grouping, we did not find a clear elbow (Supplementary Figure 4A). In the participant clustering, the curve at *K'* = 4 resembled an elbow (Supplementary Figure 4B); however, it lacked objectivity.
- **Calinski–Harabasz (CH) criterion**: This criterion was inspired by the *F*-statistic of the analyses of variance to test the significance of differences between groups. The CH criterion for each *K* was defined as:

$CH\left( K \right)=\left( \frac{S_{b}\left( K \right)}{K-1} \right)/\left( \frac{S_{w}\left( K \right)}{N-K} \right)$(S4)

where *N* is the number of data points (11). As with the *F*-statistic significance testing, a larger $CH\left( K \right)$ indicates a better clustering solution. In feature grouping, $K=2$ was optimal according to the CH criterion, which indicated no clear clustering structure (Supplementary Figure 5A). In participant clustering, $K=4$ was optimal (Supplementary Figure 5B), similar to the results of the Elbow method.

- **Bayesian information criterion (BIC)**: This was originally developed for model comparisons of statistical inference problems (e.g., the determination of the order in polynomial regression models) and is based on the asymptotic approximation of the marginal likelihood (also known as the evidence) in Bayesian statistics (12, 13). K-means clustering can be interpreted as an approximation of the statistical inference of the following mixture of Gaussian distributions:

$P(i;\theta)=g_{i}; P\left( x | i;\theta\right)=\left( 2\pi\right)^{-p/2}\left| \Sigma_{i} \right|^{-1/2}\exp\left\{ -\frac{1}{2}\left( x-\mu_{i} \right)^{T}\Sigma_{i}^{-1}\left( x-\mu_{i} \right) \right\}$ (S5)

where $\theta=\left\{ g_{i},\mu_{i},\Sigma_{i} \right\}_{i=1}^{K}$ are the model parameters, $g_{i}\in\left[ 0,1 \right]$ is the mixing rate, and $\mu_{i}\in\mathfrak{R}^{p}$ and $\Sigma_{i}\in\mathfrak{R}^{p\times p}$ are the mean vector and the covariance matrix of the *i*-th Gaussian distribution, respectively. Assuming that $\Sigma_{i}$ is restricted to a diagonal matrix, the maximized value of the likelihood function of this model is given by:

$\hat{L}=P\left( x,i;\hat{\theta} \right)=P\left( i;\hat{\theta} \right)P\left( x|i;\hat{\theta} \right) ($S6)

Herein, $\hat{\theta}=\left\{ \hat{g}_{i},\hat{\mu}_{i},\hat{\Sigma}_{i} \right\}_{i=1}^{K}$ is given by:

$\hat{g}_{i}=\frac{\left| C_{i} \right|}{N}; \hat{\mu}_{i}=\frac{1}{\left| C_{i} \right|}\sum_{x\in C_{i}} x; \hat{\Sigma}_{i}=\mathrm{diag}\left\{ \frac{1}{\left| C_{i} \right|}\sum_{x\in C_{i}} \left( x-\hat{\mu}_{i} \right)\left( x-\hat{\mu}_{i} \right)^{T} \right\} ($S7)

where $\mathrm{diag}\left\{ \cdot\right\}$ is an operator for the matrix to retain the diagonal elements as they are and set all off-diagonal elements to zero. Using the likelihood function, BIC is defined as:

$BIC\left( K \right)=-2\ln\hat{L}+m\ln N ($S8)

where $m=K(2p+1)$ is the number of parameters to be estimated from the data. A lower BIC indicates a better *K*. According to the BIC, *K* = 3 and *K'* = 2 were optimal for the feature grouping and participant clustering, respectively (Supplementary Figure 6).

- **Akaike information criterion (AIC)**: This was developed for the same purpose as the BIC. However, it was derived according to the theoretical estimate of the prediction error of the model. Because of the difference in the objective function for the model comparison, AIC was changed to:

$AIC\left( K \right)=-2\ln\hat{L}+2m ($S9)

where *m* is the same as that in the BIC (see 14, 15). According to the AIC, *K* = 4 and *K'* = 7 were optimal for the feature grouping and participant clustering, respectively (Supplementary Figure 7).

In summary, the optimal *K* varied from 2 to 4 depending on the criteria. This was also true for *K'*, and the optimal *K'* varied from 2 to 7. To preserve the rich information embedded in the high-dimensional observations as much as possible, we selected the largest *K* and *K'* in these ranges, which resulted in our focus on the AIC.

# Supplementary tables

**Supplementary Table 1.** Schedule of the assessment of delirium symptoms and risk factors.

|  | **Study period** | | | | | | | | |
| --- | --- | --- | --- | --- | --- | --- | --- | --- | --- |
|  | **Enrollment** | **Baseline** | **Operation** | **Postoperation** | | | | | |
| **Time point** |  | Day  −1 |  | Day 0 | Day 1 | Day 2 | Day 3 | Day 4 | Day 5 |
| **Assessment** |  |  |  |  |  |  |  |  |  |
| DSM-5 | x | x |  | x | x | x | x | x | x |
| DRS-R-98 |  |  |  |  | x | x | x | x | x |
| MMSE | x |  |  |  |  |  |  |  |  |
| HADS-A |  | x |  |  |  |  |  |  |  |
| BZD | x | x |  |  |  |  |  |  |  |
| Anesthesia type |  |  | x |  |  |  |  |  |  |
| Operative duration |  |  | x |  |  |  |  |  |  |

DSM-5 = Diagnostic and Statistical Manual of Mental Disorders, Fifth Edition; DRS-R-98 = Japanese version of the Delirium Rating Scale-Revised-98; MMSE = Mini-Mental State Examination; HADS-A = Hospital Anxiety and Depression Scale-Anxiety; BZD = benzodiazepine.

**Supplementary Table 2.** The 13 severity items of the Delirium Rating Scale-Revised-98.

| **No.** | **Severity item** | **Abbreviation** | **Score range** |
| --- | --- | --- | --- |
| 1 | Sleep–wake cycle disturbance | Insomnia | 0–3 |
| 2 | Perceptual disturbances | Perceptual disturbances | 0–3 |
| 3 | Delusions | Delusions | 0–3 |
| 4 | Lability of affect | Affective lability | 0–3 |
| 5 | Language | Language | 0–3 |
| 6 | Thought process | Thought | 0–3 |
| 7 | Motor agitation | Agitation | 0–3 |
| 8 | Motor retardation | Retardation | 0–3 |
| 9 | Orientation | Orientation | 0–3 |
| 10 | Attention | Attention | 0–3 |
| 11 | Short-term memory | STM | 0–3 |
| 12 | Long-term memory | LTM | 0–3 |
| 13 | Visuospatial ability | Visuospatial  ability | 0–3 |
| **Total Severity Score** | | | 0–39 |

Each item is rated from 0 (none or normal) to 3 (most severe). Their sum constitutes the total severity scale score, for which the optimal cutoff score to discriminate between the delirium and non-delirium groups was 10.0 (resulting in 98% sensitivity and 91% specificity) (16). The abbreviation column lists the shorthand notations for each item. STM = short-term memory; LTM = long-term memory.

# Supplementary figures


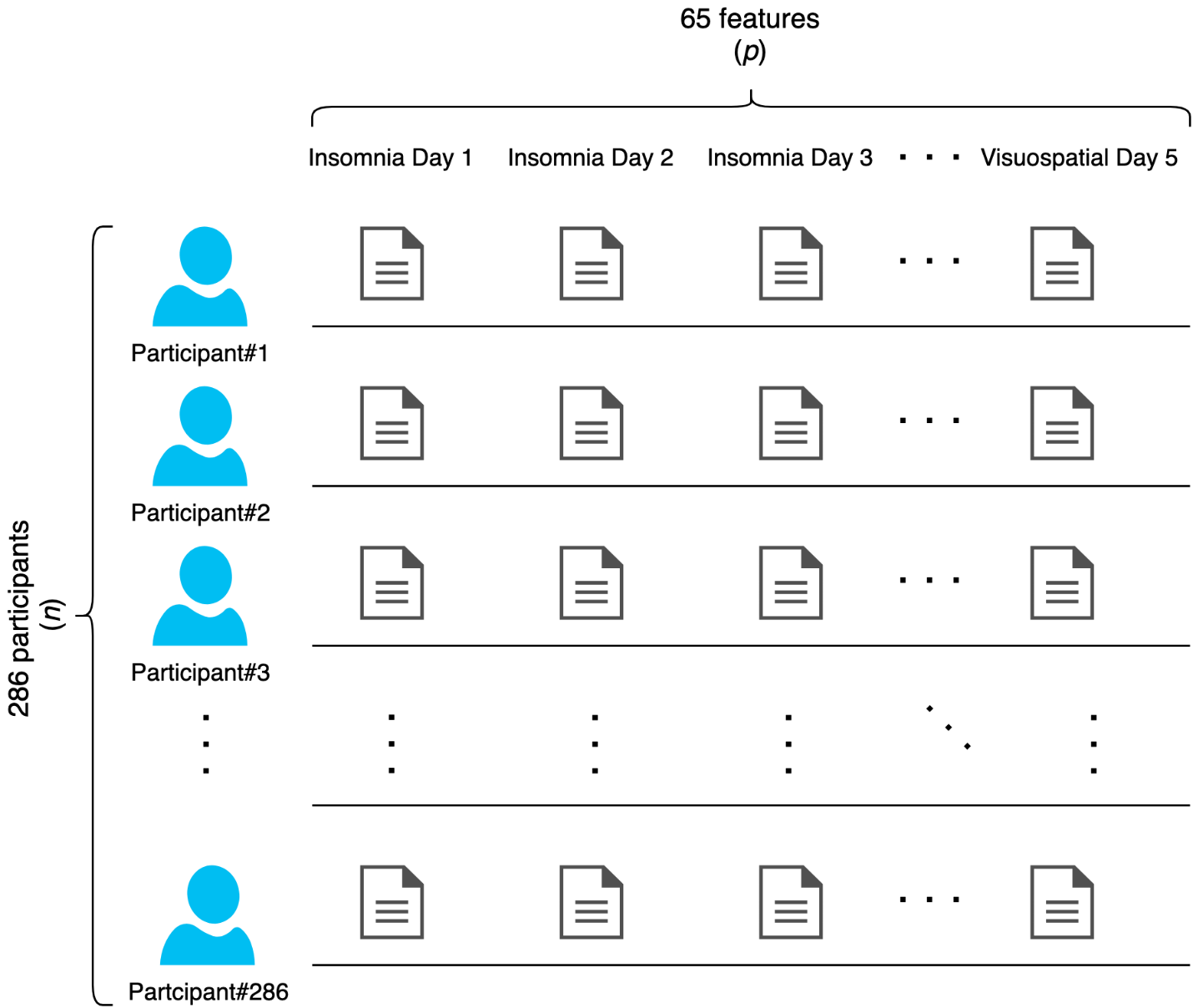


**Supplementary Figure 1.** Format of the original data matrix analyzed in this study. The rows represent participants and the columns represent the Delirium Rating Scale-Revised-98 items assessed on different days.

**Supplementary Figure 2.** Workflow of our data analysis. The original data matrix shown in Supplementary Figure 1 was analyzed using the following procedure: 1) The columns of the matrix were partitioned into mutually exclusive groups using K-means clustering, and the number of columns (i.e., the feature dimensionality) was reduced using within-group averaging (PCA was applied to the K-means clustering result just for visualization); 2) The rows of the resulting matrix (the processed data matrix) were partitioned using K-means clustering to assign the participants to mutually exclusive clusters (PCA was again applied to the second K-means clustering result just for visualization); 3) Finally, post hoc group comparison among the participant clusters was performed in terms of various scores associated with background characteristics and feature groups obtained in Step 1). Figure and table numbers indicate the locations where the corresponding results are shown. PCA = principal component analysis.


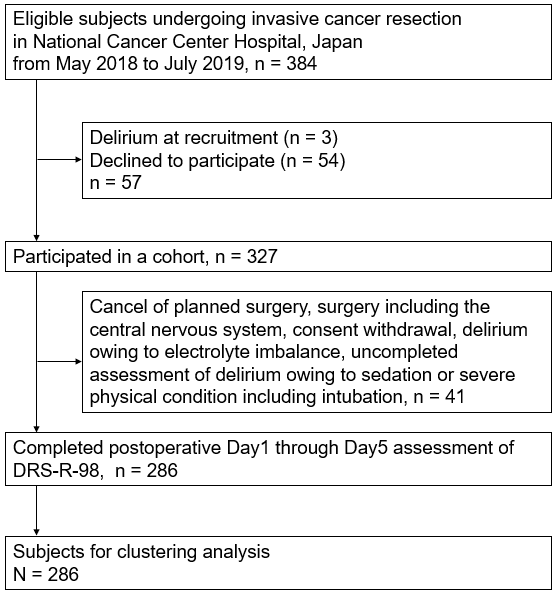


**Supplementary Figure 3.** Flow diagram of the study from enrollment to data analysis. DRS-R-98 = Delirium Rating Scale-Revised-98.

(**A**) For feature grouping (**B**) For participant clustering

**Supplementary Figure 4.** Distortion as a function of the number of clusters *K.* WCSS = within-cluster sum of squares.

(**A**) For feature grouping (**B**) For participant clustering

**Supplementary Figure 5.** The Calinski–Harabasz criterion as a function of the number of clusters *K.*

(**A**) For feature grouping (**B**) For participant clustering

**Supplementary Figure 6.** The Bayesian information criterion (BIC) as a function of the number of clusters *K.*

(**A**) For feature grouping (**B**) For participant clustering

**Supplementary Figure 7.** The Akaike information criterion (AIC) as a function of the number of clusters *K.*

**Supplementary Figure 8.** Heatmap to visualize the difference in each grouped feature (Table 2) along 65 dimensions from the 13 subscales of the Delirium Rating Scale-Revised-98 during the 5 postoperative days. The labels in the horizontal axis refer to feature groups: Mixed motor; Cognitive and higher-order thinking domain with perceptual disturbance and thought content abnormalities (abbreviated as Cognitive); Acute and temporal response (abbreviated as Acute); and Sleep–wake cycle disturbance (abbreviated as Sleep rhythm).

**Supplementary Figure 9.** Heatmap visualizing the distribution of each cluster for each grouped feature. The rows represent the participants, and the columns represent the grouped features: Mixed motor; Cognitive and higher-order thinking domain with perceptual disturbance and thought content abnormalities (abbreviated as Cognitive); Acute and temporal response (abbreviated as Acute); and Sleep–wake cycle disturbance (abbreviated as Sleep rhythm). The bold horizontal line indicates the border of the cluster. Colors closer to blue indicate higher values of the grouped feature, whereas those closer to green indicate lower values. The color patterns generally vary between clusters. For example, cluster 6 shows high scores only in the sleep rhythm feature group, whereas cluster 5 shows high scores in the hyperactive feature group as well as the sleep rhythm feature group.


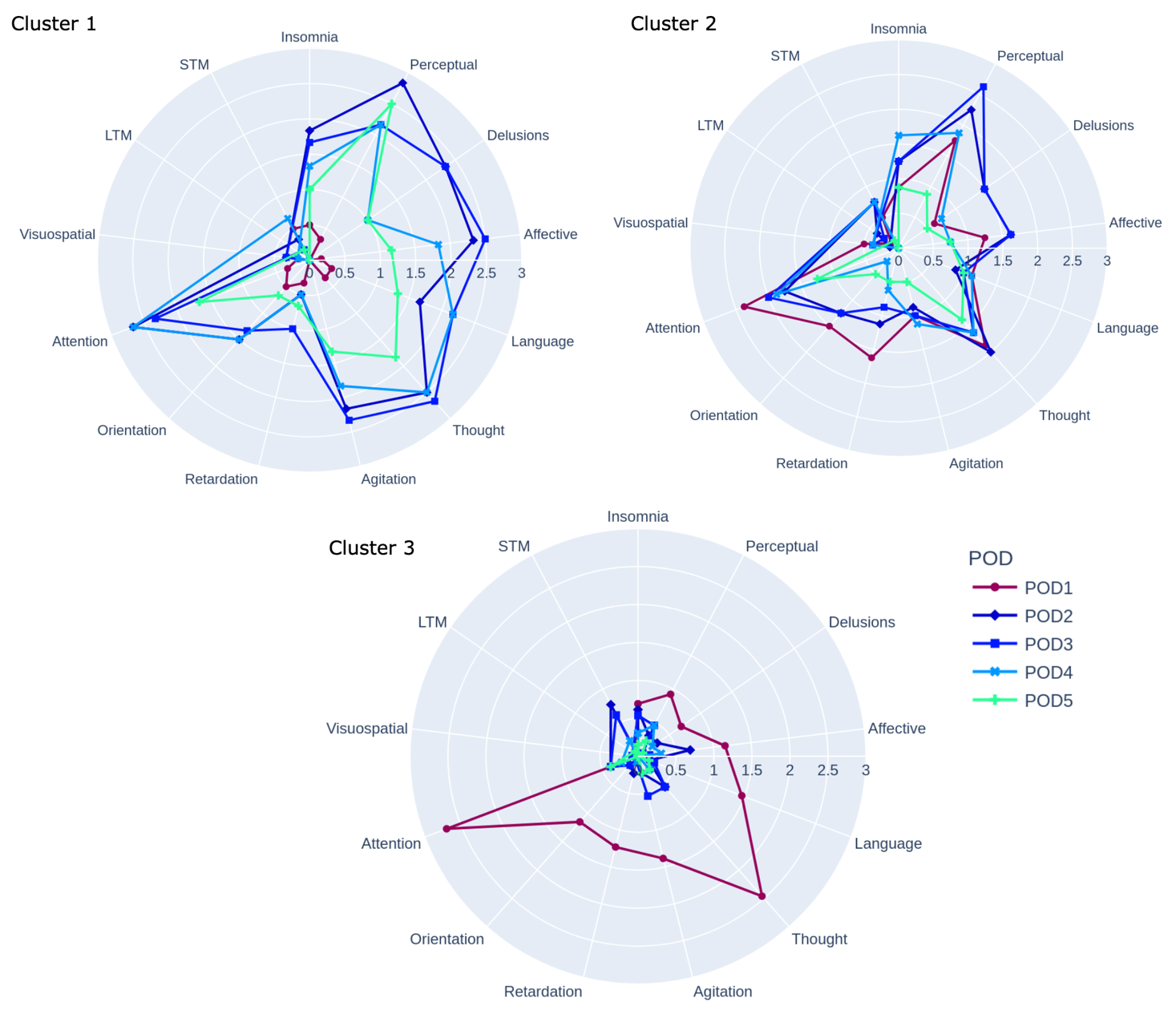
(Continued on the next page)


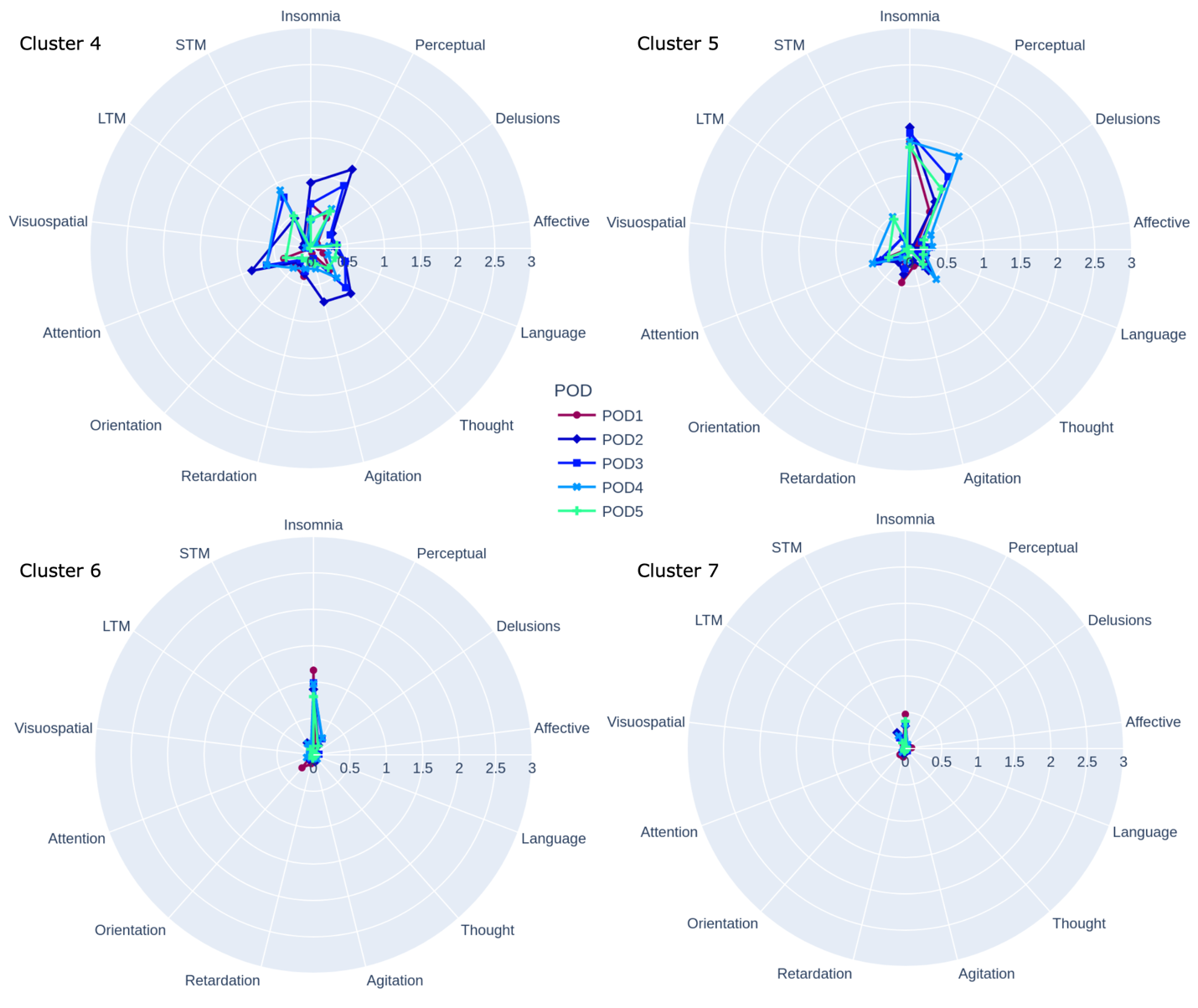


**Supplementary Figure 10.** Radar chart visualizing the mean of each cluster for all 13 types of features. The first three panels (on the previous page) show the charts for clusters 1, 2, and 3, which are the delirium clusters. The remaining four panels (on this page) show the charts for clusters 4 and 5, which are subsyndromal delirium clusters, and clusters 6 and 7, which are non-delirium clusters. STM = short-term memory; LTM = long-term memory; POD = postoperative day.

# Supplementary references

1. Hartigan JA, Wong MA. Algorithm AS 136: a K-Means Clustering Algorithm. J R Stat Soc. Series C: Appl Stat. (1979) 28:100-8. doi.org/10.2307/2346830

2. Botía JA, Vandrovcova J, Forabosco P, Guelfi S, D'Sa K, United Kingdom Brain Expression Consortium. An additional k-means clustering step improves the biological features of WGCNA gene co-expression networks. BMC Syst Biol (2017) 11:47. doi: 10.1186/s12918-017-0420-6

3. de Souto M, Costa I, Araujo D, Ludermir T, Schliep A. Clustering cancer gene expression data: a comparative study. BMC Bioinform (2008) 9:497. doi: 10.1186/1471-2105-9-497

4. Quackenbush J. Computational analysis of microarray data. Nat Rev Genet (2001) 2:418-27. doi: 10.1038/35076576

5. Malav A, Kadam K, Kamat P. Prediction of heart disease using k-means and artificial neural network as hybrid approach to improve accuracy. Int J Eng Sci Technol (2017) 9:3081-5. doi: 10.21817/ijet/2017/v9i4/170904101

6. Mahajan P, Sharma A. Role of k-means algorithm in disease prediction. Int J Comput Sci Eng (2016) 5:16216-7. doi: 10.18535/Ijecs/v5i4.28

7. Zakharov K. Application of k-means clustering in psychological studies. Quant Meth Psych (2016) 12:87-100. doi: 10.20982/tqmp.12.2.p087

8. Clatworthy J, Buick D, Hankins M, Weinman J, Horne R. The use and reporting of cluster analysis in health psychology: a review. Br J Health Psychol (2005) 10:329-58. doi: 10.1348/135910705X25697

9. Syakur MA, Khotimah BK, Rochman EMA, Satoto BD. Integration k-means clustering method and elbow method for identification of the best customer profile cluster. IOP Conf Ser: Mater Sci Eng (2018) 336:012017. doi: 10.1088/1757-899X/336/1/012017

10. Marutho D, Hendra Handaka S, Wijaya E, Muljono. The determination of cluster number at k-mean using elbow method and purity evaluation on headline news. In: 2018 International Seminar on Application for Technology of Information and Communication; 2018 Sept 21-22; Semarang, Indonesia. IEEE (2018). p. 533-8.

11. Calinski T, Harabasz J. A dendrite method for cluster analysis. Commun Stat (1974) 3:1-27. doi: 10.1080/03610927408827101

12. Kodinariya TM, Makwana PR. Review on determining number of cluster in k-means clustering. Int J Adv Res Comput Sci Manag Stud (2013) 1:90-5.

13. Schwarz G. Estimating the dimension of a model. Ann Stat (1978) 6:461-4.

14. Bozdogan H. Model selection and Akaike’s information criterion (AIC): the general theory and its analytical extensions. Psychometrika (1987) 52:345-70. doi: 10.1007/BF02294361

15. Bozdogan H. Akaike’s information criterion and recent developments in information complexity. J Math Psychol (2000) 44:62-91. doi: 10.1006/jmps.1999.1277

16. Trzepacz PT, Mittal D, Torres R, Kanary K, Norton J, Jimerson N. Validation of the Delirium Rating Scale-Revised-98: Comparison with the delirium rating scale and the cognitive test for delirium. J Neuropsychiatry Clin Neurosci (2001) 13:229-42. doi: 10.1176/jnp.13.2.229. Erratum in: J Neuropsychiatry Clin Neurosci (2001) 13(3):433. PMID: 11449030
